# Pretreatment body mass index predicts survival among patients administered nivolumab monotherapy for pretreated non‐small cell lung cancer

**DOI:** 10.1111/1759-7714.14417

**Published:** 2022-04-08

**Authors:** Hisao Imai, Erika Naito, Ou Yamaguchi, Kosuke Hashimoto, Hidetoshi Iemura, Yu Miura, Ayako Shiono, Atsuto Mouri, Kyoichi Kaira, Kunihiko Kobayashi, Hiroshi Kagamu

**Affiliations:** ^1^ Department of Respiratory Medicine Comprehensive Cancer Center, International Medical Center, Saitama Medical University Hidaka Saitama Japan

**Keywords:** body mass index, carcinoma, glasgow prognostic score, nivolumab, non‐small‐cell lung, performance status, survival

## Abstract

**Background:**

Biomarker assessments for nivolumab monotherapy efficacy in previously treated patients with non‐small cell lung cancer (NSCLC) remain unclear. We evaluated whether body mass index (BMI) and Glasgow prognostic score (GPS) are useful for assessing the efficacy of nivolumab alone as a second‐line treatment in patients with pretreated NSCLC.

**Methods:**

Data of 99 patients treated with second‐line nivolumab monotherapy for NSCLC between January 2016 and December 2019 were evaluated for prognostic values of BMI and GPS to assess their usefulness in predicting progression‐free survival (PFS) and overall survival (OS).

**Results:**

The Eastern Cooperative Oncology Group‐performance status (PS) independently predicted the second‐line nivolumab monotherapeutic effect; good PS (0–1) correlated with significantly longer PFS (4.3 vs. 1.9 months, log‐rank; *p* = 0.0004) and OS (17.7 vs. 4.6 months, log‐rank; *p* < 0.0001) than poor PS. BMI independently predicted survival, with high BMI (≥22.1 kg/m^2^) associated with significantly longer OS (19.1 vs. 8.5 months, log‐rank; *p* = 0.0023) than low BMI (<22.1 kg/m^2^). However, GPS showed no significant difference for PFS or OS.

**Conclusion:**

Among patients with NSCLC treated with nivolumab monotherapy as second‐line treatment, PS was significantly correlated with both PFS and OS and BMI with OS. Thus, BMI could be a useful predictor of survival in these patients.

## Graphical abstract

BMI independently predicted survival, with high BMI (≥22.1 kg/m^2^) associated with significantly longer OS (19.1 vs. 8.5 months, log‐rank; *p* = 0.0023) than low BMI (<22.1 kg/m^2^). Our findings demonstrate that BMI could be a useful predictor of survival in NSCLC patients treated with nivolumab monotherapy as second‐line treatment.

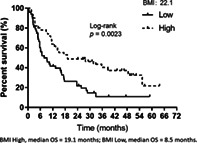

## INTRODUCTION

Lung cancer has the highest mortality rate worldwide, and non‐small cell lung cancer (NSCLC) accounts for the majority (approximately 85%–90%) of all lung cancers.^1^ Several randomized phase III trials revealed that in patients with NSCLC whose disease has progressed while on platinum‐based chemotherapy, treatment with programmed cell death‐1 (PD‐1), or programmed cell death‐ligand‐1 (PD‐L1) blockade improved overall survival (OS) compared with standard chemotherapy.^2^, ^3^, ^4^, ^5^ Thus, nivolumab monotherapy is regarded as a standard second‐line treatment for patients with NSCLC with disease progression after first‐line chemotherapy.

In previous reports, body mass index (BMI) has been used as a prognostic indicator for various cancers.^6^, ^7^, ^8^, ^9^ In patients with NSCLC treated with immune checkpoint inhibitors (ICIs), the presence of sarcopenia has reportedly had a negative prognostic effect.^6^ In addition, BMI has reportedly been associated with ICI‐related therapy outcomes in solid malignancies such as melanoma, renal cell carcinoma, and NSCLC.^7^ To date, reports on the association between BMI and Glasgow prognostic score (GPS) and the therapeutic efficacy of nivolumab monotherapy as a second‐line treatment for NSCLC are limited. A relationship between BMI and the effect of ICIs in NSCLC has recently been reported.^8^ When the BMI cutoff was set at 22 kg/m^2^ (categorized as high and low BMI groups), there was no statistically significant difference in measures of treatment efficacy, such as progression‐free survival (PFS) or OS, between the high and low BMI cohorts of NSCLC having a high PD‐L1 expression (≥50%) who received first‐line pembrolizumab monotherapy. However, when patients with NSCLC received ICI monotherapy (nivolumab/pembrolizumab/atezolizumab) as a second‐ or subsequent‐line therapy, treatment efficacy was significantly better in patients with a higher BMI than in patients with a lower BMI. Furthermore, using a BMI cutoff value of 21.4 kg/m^2^, BMI independently predicted survival because patients with high BMIs (≥21.4 kg/m^2^) had significantly longer OS (not reached vs. 14.1 months, *p* = 0.006) than patients with low BMIs (<21.4 kg/m^2^) and among patients with NSCLC and high PD‐L1 expression (≥50%) who were administered first‐line pembrolizumab monotherapy.^9^ Thus, the association between BMI and the therapeutic effect of ICIs in NSCLC is not fully understood.

Additionally, many patients with NSCLC are diagnosed at an advanced, inoperable disease stage, and they continually experience systemic inflammatory reactions (SIR) and weight loss, which reportedly have a significant impact on cancer cachexia.^10^, ^11^ Thus, prognosis attributable to cancer is assessed using an SIR‐based scoring system such as the GPS, which comprises albumin concentrations and serum C‐reactive protein (CRP) levels^10^ and is an independent prognostic factor for NSCLC.^12^, ^13^, ^14^, ^15^, ^16^, ^17^, ^18^, ^19^, ^20^, ^21^ Although the relationship between the GPS and the therapeutic effects of various types of ICIs with various lines of treatment for NSCLC has been reported in several studies,^9^, ^21^, ^22^ few reports have evaluated the relationship between GPS and effect of nivolumab monotherapy as a second‐line treatment of NSCLC. In addition, we recently reported that in patients with high PD‐L1 expression who received pembrolizumab monotherapy as a first‐line treatment for NSCLC, GPS was significantly correlated with both PFS and OS and BMI was significantly correlated with OS, which could be used to predict outcomes in these patients.^9^ We sought to determine whether these results hold true for patients with NSCLC who received nivolumab monotherapy as a second‐line therapy. Therefore, we evaluated whether BMI and GPS could predict survival for nivolumab alone as a second‐line treatment in patients with pretreated NSCLC.

## METHODS

### Patients

In the current study, we retrospectively evaluated 99 consecutive patients with advanced or metastatic NSCLC without driver gene mutation/translocation who received platinum‐based combination chemotherapy as the first‐line treatment followed by nivolumab monotherapy as a second‐line treatment after disease progression between January 2016 and December 2019. The pathological diagnosis of NSCLC was classified according to the 2015 World Health Organization Classification of Tumors, and the disease stage was assessed using version eight of the Tumor‐Node‐Metastasis (TNM) staging classification system. The inclusion criteria for enrolled patients were as follows: (1) histologically‐ or cytologically‐confirmed NSCLC, (2) platinum‐based combination chemotherapy treated with first‐line chemotherapy, and (3) nivolumab treatment as second‐line chemotherapy. The included patients did not receive any ICIs prior to nivolumab monotherapy as the second‐line treatment. Nivolumab, intravenously administered at 3 mg/kg or 240 mg/day every 2 weeks, was repeated until the disease progressed, unacceptable toxicity was observed, or the patient refused treatment. For first‐line and subsequent‐line therapeutic regimens, the regimen was chosen at the discretion of the attending physician. Patients' electronic medical record data were viewed and data on patient backgrounds and treatment responses to second‐line nivolumab were collected retrospectively. The study design was approved by the Institutional Ethics Committee of the International Medical Center, Saitama Medical University (approval number: 20–247). The protocol was performed in accordance with the 1964 Declaration of Helsinki (revised in 2008). The requirement for written informed consent was waived by the Ethics Committee because of the retrospective nature of the study; however, the patients were provided with an opt‐out opportunity.

### Evaluation of treatment efficacy

Serum CRP and albumin concentration levels were measured on the day of or day before initiation of nivolumab monotherapy. The GPS was classified into three groups according to the combination of CRP and albumin as follows: GPS 0, 1, and 2 included patients with a CRP <1.0 mg/dl and albumin >3.5 mg/dl; CRP only was increased or albumin only was decreased; and CRP was ≥1.0 mg/dl and albumin level <3.5 mg/dl, respectively. BMI calculated before the start of nivolumab treatment was defined as weight (kg) divided by height (m) squared. Patients were classified into high and low BMI groups, as defined by the receiver operating characteristic (ROC) curve: low (<22.1 kg/m^2^) and high (≥22.1 kg/m^2^) BMI. The appropriate cutoff value differentiating the high and low BMI groups, based on the ROC curve analysis for OS, was 22.1 kg/m^2^ (area under the curve [AUC]: 0.582; sensitivity: 60.7%; specificity: 41.6%).

Tumor response was evaluated as the best overall response and maximum tumor shrinkage. Radiographic tumor responses were evaluated using the Response Evaluation Criteria in Solid Tumors (RECIST) version 1.1.^23^ PFS was defined as the time from the initial nivolumab monotherapy to disease progression or death. OS was defined as the time from the initial nivolumab monotherapy to death or was censored on the date of the last follow‐up.

### Statistical analysis

For statistical analysis, we used Fisher's exact test and Welch's *t*‐test for categorical and continuous variables, respectively. Cox proportional hazards models with stepwise regression were applied to identify factors predicting PFS and OS, and the results are presented as hazard ratios (HRs) and 95% confidence intervals (CIs). The Kaplan–Meier method was used to estimate survival as a function of time, and survival differences were analyzed using the log‐rank test. We performed univariate and multivariate logistic regression analyses according to the different outcome variables. The statistical significance level was set at a *p*‐value ≤0.05. All statistical analyses were performed using the JMP software for Windows, version 11.0 (SAS Institute).

## RESULTS

### Patient backgrounds and therapeutic efficacy

Table 1 presents the characteristics of the 99 patients including 77 men (77.8%) and 22 women (22.2%), with a median age of 69 years (range, 31–80 years). The Eastern Cooperative Oncology Group (ECOG)‐performance status (PS) was 0–1 in 77 patients (77.8%) and 2–3 in 22 patients (22.2%). Adenocarcinoma accounted for 56 of the 99 patients (56.6%). Eighty‐two patients (82.8%) had stage III–IV disease. The number of patients not evaluated for PD‐L1 expression was 82 (82.8%). All cases were wild‐type, negative, or unknown for driver gene mutation/translocation status. The median BMI was 22.0 (range, 14.3–33.7 kg/m^2^). The median number of nivolumab administration cycles was four (range, 1–97). The responses to nivolumab monotherapy among all patients were identified as follows: complete response (*n* = 1), partial response (*n* = 18), stable disease (*n* = 30), and progressive disease (*n* = 40). Thus, the overall response rate was 19.2% (95% CI: 11.4–26.9), and the disease control rate was 49.5% (95% CI: 39.6–59.3).

**TABLE 1:** Patient baseline characteristics

Characteristics	Patients (n)
Total number of patients (*n*)	99
Sex	
Men/women	77/22
Median age at initiation of nivolumab (years) [range]	69 (31–80)
Performance status (PS)	
0/1/2/3/4	36/41/15/7/0
Clinical disease stage at diagnosis	
III/IV/postoperative recurrence	16/66/17
Smoking history	
Current or former/never	84/15
Histological classification	
Adenocarcinoma/squamous cell carcinoma/others	56/25/18
PD‐L1 tumor proportion score (%)	
≥1/0/untested	3/14/82
Driver gene mutation/translocation	
*EGFR*/*ALK*/wild‐type, negative, or unknown	0/0/99
BMI (kg/m^2^)	
Median [range]	22.0 (14.3–33.7)
Prior radiation therapy	
Yes/no	36/63
Administration cycles of nivolumab	
Median (range)	4 (1–97)
Treatment response	
CR	1
PR	18
SD	30
PD	40
NE	10
Response rate (%) (95% CI)	19.2 (11.4–26.9)
Disease control rate (%) (95% CI)	49.5 (39.6–59.3)
Laboratory data (median) [range]	
Albumin (g/dl)	3.7 (2.3–4.7)
CRP (mg/dl)	0.773 (0.01–25.983)

Abbreviations: ALK, anaplastic lymphoma kinase; BMI, body mass index; CI, confidence interval; CR, complete response; CRP, C‐reactive protein.; EGFR, epidermal growth factor receptor; NE, not evaluated; PD, progressive disease; PD‐L1, programmed death‐ligand 1; PR, partial response; PS, performance status; SD, stable disease.

### Comparative analysis of the GPS and BMI

The patient characteristics according to GPS and BMI are shown in Table 2. The GPS values at the initiation of nivolumab administration revealed a GPS of 0–1 in 72 patients and a GPS of 2 in 27 patients. There were significant differences (*p* < 0.05) in ECOG‐PS, clinical stage at diagnosis, prior radiotherapy, albumin levels, and CRP levels between GPS categories (0–1 vs. 2). At the start of nivolumab administration, low and high BMI were reported in 48 and 51 patients, respectively. The administration cycles of nivolumab were significantly different (*p* < 0.05) between the BMI categories.

**TABLE 2:** Results of patient baseline characteristics according to the Glasgow prognostic score and body mass index

	GPS			BMI		
Variables	0–1	2	*p*‐value	Low (<22.1)	High (≥22.1)	*p*‐value
Patients (n)	72	27		48	51	
Baseline characteristics						
Sex						
Men/women	55/17	22/5	0.78	38/10	39/12	0.81
Median age at treatment (years) (range)	69 (31–80)	69 (50–79)	0.46^a^	67.5 (50–79)	70 (31–80)	0.25^a^
Performance status (PS)						
0–1/3–4	63/9	14/13	**0.0003**	34/14	43/8	0.14
Smoking history						
Yes/no	62/10	22/5	0.54	42/7	42/9	0.78
Histological classification						
Adenocarcinoma/nonadenocarcinoma	43/29	13/14	0.36	26/22	30/21	0.68
Clinical stage at diagnosis						
III–IV/postoperative recurrence	55/17	27/0	**0.0051**	42/6	40/11	0.29
BMI (kg/m^2^)						
Median (range)	22.3 (14.3–33.7)	21.7 (15.4–27.7)	0.06^a^	20.1 (14.3–22.0)	24.0 (22.1–33.7)	‐
Prior radiation therapy						
Yes/no	19/53	17/10	**0.0011**	20/28	16/35	0.30
Administration cycles of nivolumab						
Median (range)	5 (1–97)	4 (1–36)	0.05^a^	4 (1–69)	5 (1–97)	**0.04** ^a^
Treatment response						
CR	1	0		0	1	
PR	11	7		9	9	
SD	26	4		12	18	
PD	27	13		22	18	
NE	7	3		5	5	
Response rate (%) (95% CI)	16.6 (8.0–25.2)	25.9 (9.3–42.4)	0.39	18.3 (7.7–29.7)	20.0 (8.7–30.5)	>0.99
Disease control rate (%) (95% CI)	52.7 (41.2–64.3)	40.7 (22.2–59.2)	0.36	44.8 (29.7–57.7)	54.0 (41.7–68.5)	0.31
Laboratory data						
Median (range)						
Albumin (g/dl)	3.9 (3.2–4.7)	2.9 (2.3–3.4)	**<0.0001** ^a^	3.6 (2.3–4.7)	3.8 (2.7–4.7)	0.05^a^
CRP (mg/dl)	0.411 (0.01–7.787)	5.162 (1.029–25.983)	**<0.0001** ^a^	1.17 (0.01–23.789)	0.754 (0.028–25.983)	0.49^a^

*Note*: *p‐*values in bold are statistically significant (*p* < 0.05).

Abbreviations: BMI, body mass index; CI, confidence interval; CR, complete response; CRP, C‐reactive protein; GPS, Glasgow Prognostic Score; NE, not evaluated; PD, progressive disease; PR, partial response; PS, performance status; SD, stable disease.

^a^ Welch's *t*‐test.

### Analysis of survival

**FIGURE 1:**
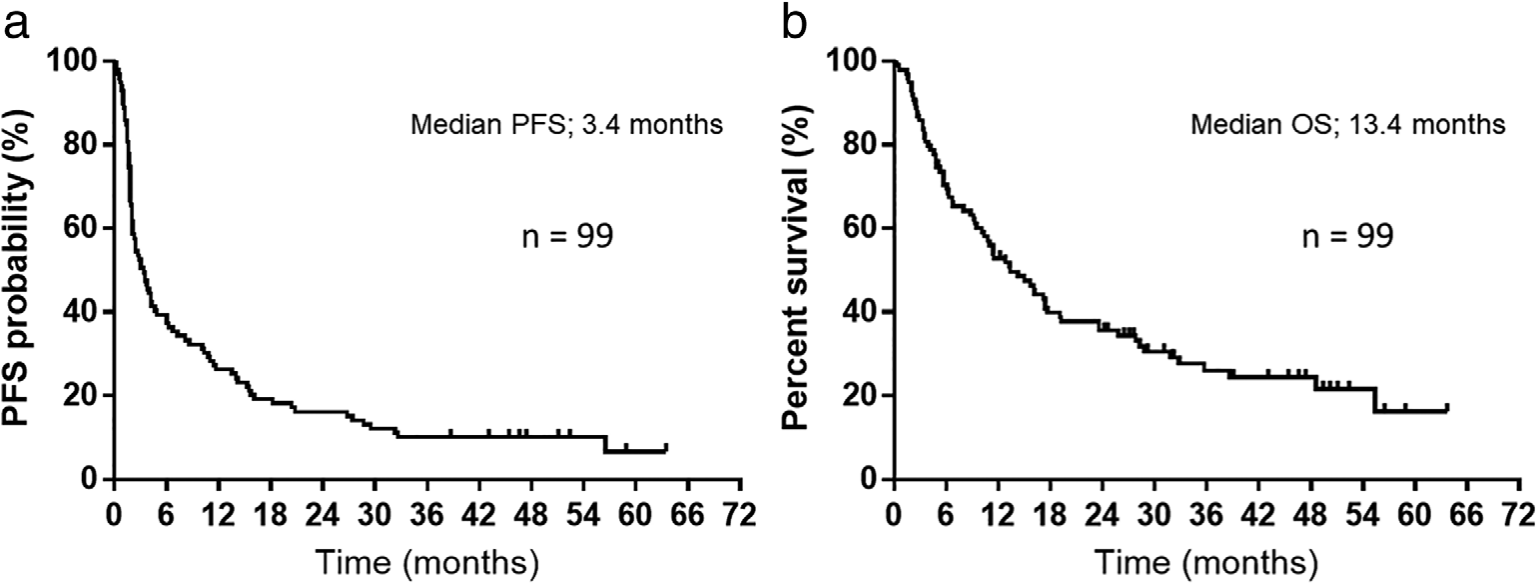
Kaplan–Meier curves for progression‐free survival (PFS) and overall survival (OS) among 99 patients who received nivolumab monotherapy as a second‐line treatment. (a) The median PFS was 3.4 months (95% confidence interval [CI] 2.1–4.7). (b) The median OS was 13.4 months (95% CI: 9.5–17.7)

**FIGURE 2:**
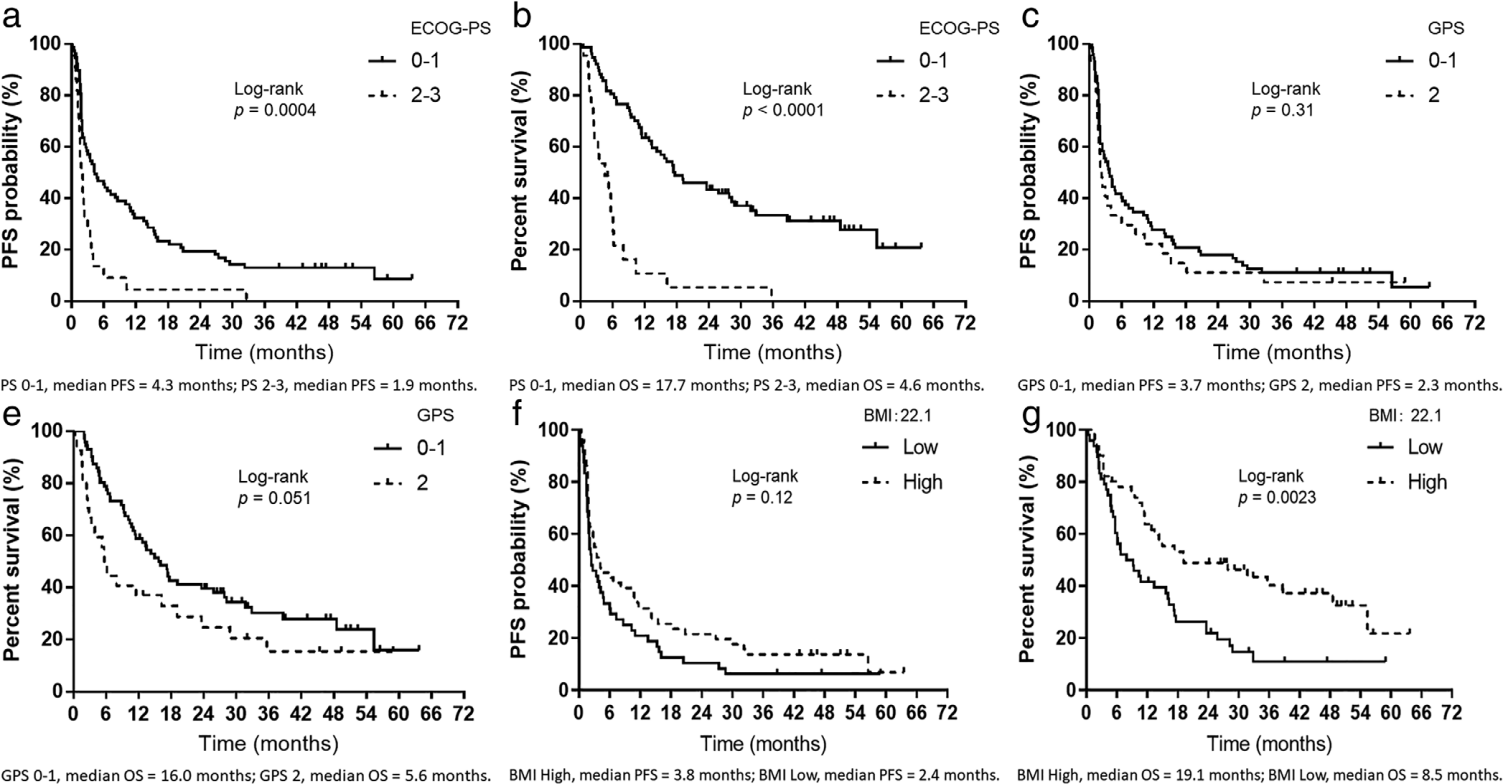
Kaplan–Meier curves for progression‐free survival (PFS) and overall survival (OS) according to the Eastern Cooperative Oncology Group (ECOG) performance status (PS), Glasgow prognostic score, and body mass index. (a) PFS according to ECOG‐PS at the initiation of nivolumab administration (Glasgow prognostic score [GPS] 0–1, median PFS = 4.3 months; GPS 2, median PFS = 1.9 months). (b) Overall survival (OS) according to ECOG‐PS at the initiation of nivolumab monotherapy (GPS 0–1, median OS = 17.7 months; GPS 2, median OS = 4.6 months). (c) PFS according to GPS at the initiation of nivolumab administration (GPS 0–1, median PFS = 3.7 months; GPS 2, median PFS = 2.3 months). (d) OS according to GPS at the initiation of nivolumab administration (GPS 0–1, median OS = 16.0 months; GPS 2, median OS = 5.6 months). (e) PFS according to BMI at the initiation of nivolumab administration (body mass index [BMI] high, median PFS = 3.8 months; BMI low, median PFS = 2.4 months). (f) OS according to BMI at the initiation of nivolumab administration (BMI high, median OS = 19.1 months; BMI low, median OS = 8.5 months)

**FIGURE 3:**
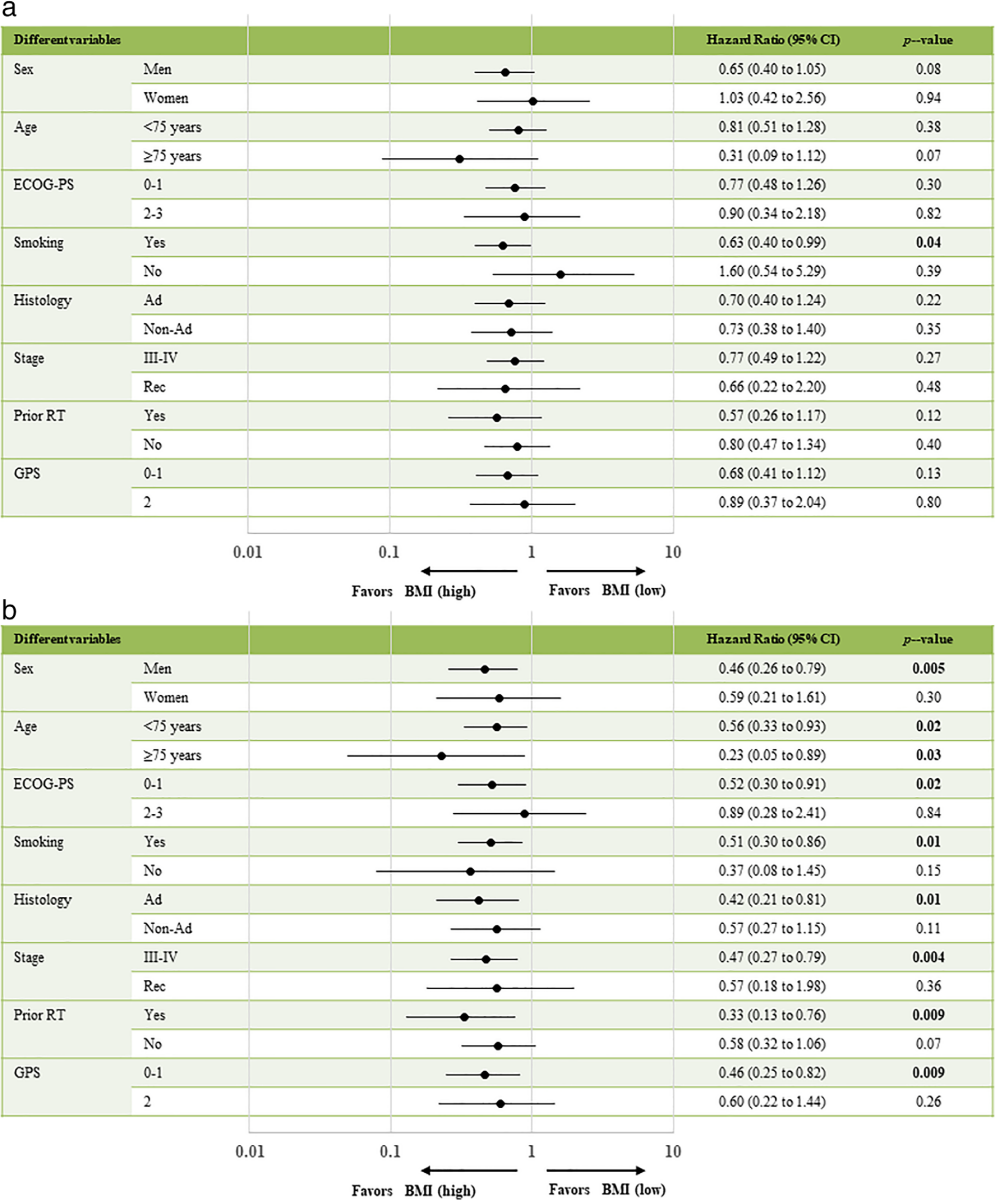
(a) Analysis of PFS and (b) OS by key clinical factors of body mass index. Ad, adenocarcinoma; BMI, body mass index; CI, confidence interval; ECOG‐PS, Eastern Cooperative Oncology Group‐performance status; GPS, Glasgow prognostic score; PFS, progression‐free survival; rec, recurrence; RT, radiotherapy

The median PFS interval was 3.4 months (95% CI: 2.1–4.7) (Figure 1a), the median OS interval was 13.4 months (95% CI: 9.5–17.7) (Figure 1b), and the median follow‐up duration was 12.7 (range, 0.3–63.7) months. Of the 99 patients included in the analysis, 72 died while 27 were still alive as of September 30, 2021, the data cutoff date. As shown in Table 3, the results of the univariate and multivariate analyses of PFS and OS are demonstrated. Univariate analyses of PFS demonstrated significant associations with ECOG‐PS. Multivariate analyses demonstrated that PFS was associated with PS (0–1 and 2–3) (HR: 0.44, *p* = 0.0027). Furthermore, significant correlations were found between ECOG‐PS and BMI in the univariate analysis of OS. Multivariate analyses revealed that OS was significantly correlated with PS (0–1 and 2–3) (HR: 0.25, *p* < 0.0001) and BMI (low/high) (HR 1.79, *p* = 0.0184). Figure 2 demonstrates the survival curves for PFS and OS, according to ECOG‐PS, GPS, and BMI; an ECOG‐PS of 0–1 correlated significantly with longer PFS and OS than an ECOG‐PS of 2–3 (both, *p* < 0.05; Figure 2a, b). There was no statistically significant difference in PFS and OS between a GPS of 0–1 and a GPS of 2 (Figure 2c,d). Although BMI was not associated with PFS (*p* = 0.12, Figure 2e), BMI was significantly associated with OS (*p* = 0.0023, Figure 2f). Furthermore, in the forest plot analysis, key clinical factors of survival (PFS or OS) by BMI subgroup (high or low) were evaluated (Figure 3a,b). As a result, men aged <75 years or ≥ 75 years, ECOG‐PS of 0–1, smokers, adenocarcinoma, stage III–IV disease, prior radiotherapy, and GPS of 0–1 were identified as significant factors of OS according to the BMI subgroups, although none were factors of PFS except for current or former smoker (Figure 3a,b).

**TABLE 3:** Univariate and multivariate analyses of progression‐free survival and overall survival

	Median PFS	Univariate analysis			Multivariate analysis			Median OS	Univariate analysis			Multivariate analysis		
Variables	(months)	HR	95% CI	*p*‐value	HR	95% CI	*p*‐value	(months)	HR	95% CI	*p*‐value	HR	95% CI	*p*‐value
Sex														
Men/women	3.0/3.4	0.97	0.60–1.65	0.92				14.3/11.9	0.94	0.55–1.71	0.84			
Age (years)														
<75/≥75	3.0/7.7	1.28	0.74–2.37	0.37				13.3/17.4	1.33	0.71–2.78	0.37			
Performance status (PS)														
0–1/2–3	4.3/1.9	0.42	0.26–0.71	**0.0016**	0.44	0.27–0.74	**0.0027**	17.7/4.6	0.22	0.13–0.39	**<0.0001**	0.25	0.14–0.44	**<0.0001**
Smoking history														
Yes/No	3.5/2.8	0.90	0.52–1.66	0.72				13.4/19.2	1.24	0.66–2.58	0.50			
Histological classification														
Adenocarcinoma/nonadenocarcinoma	3.1/3.5	1.03	0.68–1.58	0.86				15.5/11.5	0.85	0.53–1.36	0.49			
Clinical stage at diagnosis														
III–IV/postoperative recurrence	2.4/6.2	1.69	0.98–3.12	0.05				11.5/23.6	1.38	0.78–2.65	0.26			
Prior radiotherapy														
Yes/No	5.3/2.4	0.67	0.42–1.04	0.07				15.7/13.4	0.88	0.53–1.43	0.63			
GPS														
0, 1/2	3.7/2.3	0.79	0.50–1.27	0.32				16.1/5.7	0.61	0.37–1.02	0.06			
BMI (kg/m^2^)														
Low (<22.1)/high (≥22.1)	2.4/3.8	1.38	0.91–2.11	0.12	1.29	0.84–1.97	0.22	8.5/19.1	2.05	1.28–3.32	**0.0027**	1.79	1.10–2.93	**0.0184**

*Note*: The reference arms are the variables shown in the right‐sided arms. *p‐*values in bold are statistically significant (*p* < 0.05).

Abbreviations: BMI, body mass index; CI, confidence interval; GPS, Glasgow prognostic score; HR, hazard ratio; OS, overall survival; PFS, progression‐free survival; PS, performance status.

## DISCUSSION

This study evaluated the relationship between BMI and GPS and treatment survival efficacy in patients treated with nivolumab monotherapy as a second‐line treatment for NSCLC. In multivariate analyses, ECOG‐PS and BMI were independent prognostic factors for OS, suggesting that ECOG‐PS and BMI are clinically useful factors for predicting OS in patients who were treated with nivolumab monotherapy as a second‐line treatment for NSCLC. In addition, high BMI was identified as an independent predictor for OS, particularly in men aged <75 years or ≥ 75 years with ECOG‐PS of 0–1 who are smokers with adenocarcinoma, stage III–IV disease, prior radiotherapy, and a GPS of 0–1. Additionally, a high BMI was an independent predictor of OS irrespective of age.

The GPS was derived from serum CRP and albumin concentrations that can be easily measured in routine clinical practice.^10^ Although several reports demonstrate associations between the GPS and therapeutic effects of ICIs in patients with NSCLC with different treatment lines and various ICIs and levels of PD‐L1 expression,^9^, ^20^, ^21^ no significant differences between GPSs of 0–1 and 2 were observed in terms of PFS and OS in the current study. Reasons for this may include the relatively small number of patients and the influence of various factors from previous treatments since this was a study of second‐line treatments. However, the cause is still unclear, and further investigations are needed in the future. Furthermore, as shown in Figure 3b, the high BMI group had better OS than the low BMI group with GPSs of 0–1, suggesting that the high BMI group had a better prognosis with a GPS of 0–1.

In this study, ECOG‐PS and BMI were independent prognostic factors for OS in multivariate analyses. In particular, the ECOG‐PS, which is a subjective scoring system that evaluates the overall general condition of patients with cancer and is correlated independently with PFS and OS, has been a demonstrated potent prognostic factor.^24^, ^25^ The present study reaffirms PS as a strong prognostic factor, as reported in previous studies,^24^, ^25^ and suggests that our study population likely reflects the general patient population. Regarding BMI, a retrospective study of a large number of patients reported that a high BMI was associated with prolonged PFS and OS after ICI treatment in patients with advanced melanoma.^26^ Other retrospective studies on solid malignancies such as melanoma, renal cell carcinoma, and NSCLC, reported that BMI is associated with ICI efficacy.^7^ In addition, a relationship between BMI in patients with NSCLC treated with ICI and survival, such as PFS and OS, has been oberved.^8^ BMI is significantly related with the survival efficacy of ICI therapy in patients with NSCLC treated with second‐line or subsequent‐line PD‐1/PD‐L1 blockade therapy, and is reported to have better outcomes for patients with a high BMI. Table 4 summarizes the results of our study and those of previous studies that have evaluated BMI in patients with advanced NSCLC previously treated with ICIs.^8^, ^27^, ^28^, ^29^, ^30^, ^31^

**TABLE 4:** Reports of the body mass index on immune checkpoint inhibitor therapy for pretreated advanced non‐small cell lung cancer

Report	Year	Region	Study type	Sample size	Stage	Treatment	BMI cutoff	Treatment line	Outcome according to BMI HR (95% CI), *p*‐value	
									PFS	OS
Popinat et al.^27^	2019	France	Retrospective	55	IV	Nivolumab monotherapy	24.7	Pretreated (≥second line)	NR	(High/Low): NR, *p* = 0.082 OS tends to be longer
Ichihara et al.^22^	2020	Japan	Retrospective	429	Advanced, recurrence	Pembrolizumab, nivolumab, or atezolizumab monotherapy	22.0	Pretreated (≥second line)	(High/Low): 0.79 (0.64–0.98), ***p* = 0.036**	(High/Low): 0.73 (0.57–0.95), ***p* = 0.021**
Kichenadasse et al.^28^	2020	Global	Prospective	1434	IV, recurrence	Atezolizumab monotherapy	25.0	Untreated and pretreated	(High/Low): overweight, 0.89 (0.78–1.01), *p* = 0.09, obese, 0.86 (0.73–1.01), *p* = 0.09	(High/Low): overweight, 0.81 (0.68–0.95), ***p* < 0.001**, obese, 0.64 (0.51–0.81), ***p* < 0.001**
Dimitrakopoulos^29^	2020	Greece	Retrospective and prospective	112	III–IV	Pembrolizumab or nivolumab monotherapy	26.26	Untreated and pretreated (≥second‐line)	(High/Low): 0.738 (0.471–1.156), *p* = 0.160	(High/Low): 0.853 (0.507–1.436), *p* = 0.542
Takada et al.^30^	2020	Japan	Retrospective	226	IIIB–IV, recurrence	Pembrolizumab or nivolumab monotherapy	19.1	Untreated and pretreated (≥second‐ line)	(Low/High): 1.47 (1.04–2.05), ***p* = 0.0269**	(Low/High): 1.29 (1.10–2.30), ***p* = 0.0138**
Dragomir et al.^31^	2021	Romania	Retrospective	80	I–IV	Nivolumab monotherapy	25.0	Pretreated	(High/Low): 0.96 (0.96–1.91), ***p* = 0.001**	NR
Current study		Japan	Retrospective	99	III–IV, recurrence	Nivolumab monotherapy	22.1	Pretreated (second‐line only)	(Low/High): 1.29 (0.84–1.97), *p* = 0.22	(Low/High): 1.79 (1.10–2.93), ***p* = 0.0184**

*Note*: *p‐*values in bold are statistically significant (*p* < 0.05).

Abbreviations: BMI, body mass index; CI, confidence interval; HR, hazard ratio; NR, not reported; OS, overall survival; PFS, progression‐free survival.

When comparing the high and low BMI cohorts, the number of nivolumab administration cycles was statistically significantly higher in the high BMI cohort. However, the OS was significantly better in the high BMI cohort, but not the PFS. This may enhance the prognostic benefit of nivolumab monotherapy in patients with a high BMI and may also provide patients with the opportunity to receive more nivolumab cycles and an improved course of treatment after nivolumab. A study examining the association between BMI at the start of treatment and OS in 703 patients with metastatic NSCLC treated with nivolumab and pembrolizumab found that low BMI was associated with shorter OS (HR: 1.66, *p* = 0.002) and a high BMI with a longer OS (HR: 0.75, *p* = 0.039), similar to the findings of our study.^32^ Another report examined the correlation of BMI with PFS and OS in patients with melanoma using ipilimumab and showed a nonsignificant trend toward longer OS in overweight patients, (*p* = 0.056, log‐rank test; HR: 1.81, Cl: 95%: 0.98–3.33). Moreover, there was no significant difference in PFS (*p* = 0.924, log‐rank test; HR: 1.03, CI: 95%: 0.62–1.70).^33^ In patients with previously treated NSCLC, no significant difference was observed in PFS or OS between the low and high BMI groups (Table 4), while other studies reported both PFS and OS as being better in the high BMI group.^22^, ^30^ However, some reports indicate better OS in the high BMI group only, consistent with our study.^27^, ^28^ This discrepancy between PFS and OS is a common observation when assessing the efficacy of immunotherapy. Reasons for this include the difficulty in determining reliable PFS, and the possibility of residual effects of immunotherapy. Our findings are consistent with those of previous studies suggesting that high BMI is associated with improved survival with ICI therapy in various cancer types (melanoma, renal cell carcinoma, and NSCLC). Although previous reports included second‐line nivolumab monotherapy, none focused exclusively on second‐line nivolumab monotherapy. Furthermore, BMI can be easily used in daily clinical practice. BMI was an independent prognostic factor for OS as well as ECOG‐PS (Table 3) in our analysis. BMI is an objective parameter that allows for a more accurate and objective classification of patients. Therefore, BMI can be easily measured prior to the start of treatment in the clinical setting. In clinical practice, medical oncologists do not usually hesitate to initiate nivolumab monotherapy simply because of a low BMI, although they are hesitant in patients with poor PS. Therefore, these two indices (ECOG‐PS and BMI) of different dimensions need to complement each other in clinical practice, and it is reasonable to take BMI into account in clinical practice when administrating ICI therapy. In addition, the results showed that a high BMI is associated with a good prognosis, although this does not mean that obesity is acceptable for improving the prognosis of patients with NSCLC, and identification of the optimal BMI should be investigated in the future.

The pathophysiology of the positive correlation between overweight and survival after ICI treatment is currently unclear, although leptin‐mediated T cell dysfunction has been implicated.^34^ Based on this hypothesis, nivolumab, which acts as an anti‐PD‐1 antibody and inhibits the binding between PD‐L1/PD‐1 molecules, may induce better efficacy in patients with high BMI and manifest PD‐1 T cell exhaustion. However, the rationale for this remains unclear and there is a need to clarify the pathophysiology of how immunotherapy offers better prognostic effects in patients who are overweight.

There are several limitations to this study. First, this was a retrospective study that relied on subjective efficacy judgments by the treating physicians, which undeniably led to treatment response and PFS data variabilities. Second, there is no absolutely established cutoff value for BMI or laboratory data, and various cutoff values have been used in previous studies. We used ROC curves to determine the BMI cutoff value and those of previous studies for GPS. Furthermore, the BMI cutoff value for Westerners may be different from that of Japanese people, who are mostly small‐statured. Therefore, it is important to validate the clinical relevance of these cutoff values in a larger patient cohort in future. Another prognostic factor that affected survival in patients with NSCLC is weight loss before treatment, a known indicator for cachexia.^35^, ^36^, ^37^ Weight loss before or during treatment may have various effects in the therapeutic response to ICI administration. In our study, BMI was calculated using a single measurement of height and weight prior to nivolumab administration, although the role of weight loss prior to or during treatment was difficult to analyze or evaluate because of inconsistent records of weight loss prior to and during nivolumab administration. Third, the nivolumab dose was adjusted to 3 mg/kg bodyweight on some occasions, and at other times to a fixed dose of 240 mg/day. In this regard, in Japan, the dosage of nivolumab was approved to change from 3 mg/kg to 240 mg/bodyweight by the Japanese regulatory authority in August 2018, and since then the dosage of 3 mg/kg cannot be used. Therefore, patients who earlier received the 3 mg/kg dose also switched to 240 mg/bodyweight dosage during the course of therapy. A previous report has shown the equivalence of nivolumab administration at 3 mg/kg and 240 mg/bodyweight,^38^ and we do not believe that this has any impact on the current study.

In conclusion, this study confirmed ECOG‐PS as an independent prognostic factor for PFS and OS. Furthermore, BMI was an independent prognostic factor for OS. Although further studies are needed to validate these findings, the results suggest that BMI assessment may be useful in predicting the OS of patients who received nivolumab monotherapy as a second‐line treatment. Therefore, a larger study is needed to evaluate whether our results can be generalized to other second‐line ICI‐treated patient populations. Furthermore, it is unclear from the findings of the current study whether BMI can be considered a treatment effect modifier, although future large‐scale analyses of the effects of BMI subgroups in clinical trials of ICI treatments may answer this question.

## CONFLICT OF INTEREST

The authors declare that they have no conflicts of interest.

## Data Availability

The data that support the findings of this study are available from the corresponding author, HI, upon reasonable request.
